# *Military Medical Research* reviewer acknowledgement 2014-2015

**DOI:** 10.1186/s40779-016-0077-2

**Published:** 2016-05-12

**Authors:** Xiao-Bing Fu

**Affiliations:** Chinese PLA General Hospital, Beijing, China

## Abstract

The editors of *Military Medical Research* would like to thank all our reviewers who have contributed to the journal in volume 1 (2014) and 2 (2015).

**Maria das Graças Anguera**

Brazil

**Christian Bautista**

United States of America

**Eric Bernes**

Switzerland

**Zhao-xiang Bian**

Hong Kong

**Timothy Billiar**

United States of America

**Selami Çakmak**

Turkey

**Chuang Cai**

China

**Guangwen Cao**

China

**Wu-Chun Cao**

China

**Roberto Casale**

Italy

**Kevin Casey**

Afghanistan

**Theresa M. Casey**

United States of America

**Anne-Marie Chang**

United States of America

**Bohua Chen**

China

**Yao-Long Chen**

China

**Ning-Hai Cheng**

China

**Angela Chow**

Singapore

**Ahmet Yilmaz Coban**

Turkey

**Laura Coffey**

Ireland

**Le Cong**

United States of America

**Travis Craddock**

United States of America

**Robert Dahnke**

Germany

**Domenico De Berardis**

Italy

**Andrew Dwork**

United States of America

**Roberto Augusto Estrada Castañon**

Mexico

**Christiane Kruse Fæste**

Norway

**Ibrahima Soce Fall**

Senegal

**Jie Fan**

United States of America

**Yi-Qun Fang**

China

**Cheng Feng**

China

**Wolfgang Freund**

Germany

**Carlo Fremd **

Germany

**Xiao-Bing Fu**

China

**Hui Gao**

China

**Xu-Bing Gao**

China

**Angelo Gemignani**

Italy

**Yan Geng**

China

**Aayush Gupta**

India

**Marios Hadjicharalambous**

Cyprus

**John Harkness**

Australia

**Rod Hay**

United Kingdom

**Antonia Hildebrand**

Germany

**Hiroyuki Hirasawa**

Japan

**Yamashita Hiromasa**

Japan

**Lan Huang**

China

**Sha Huang**

China

**Sigurd Hystad**

Norway

**Jian-Xin Jiang**

China

**Ronald Kessler**

United States of America

**Jae-Seok Kim**

South Korea

**Young Wan Kim**

South Korea

**Luther C. Kloth**

United States of America 

**Yankaskas Kurt**

United States of America

**Bas De Laat**

Netherlands

**Xi-Lan Lai**

China

**Li Li**

China

**Jing Li**

China

**Shao-hua Li**

United States of America

**You-Sheng Li**

China

**Thomas Liesegang**

United States of America

**Ji-Long Liu**

United Kingdom

**Wei Liu**

China

**Wei-Wei Liu**

China

**Xiao-rong Liu**

China

**Xin Luo**

China

**Gang Lv**

China

**Vinay Mahishale**

India

**Luis Massuca**

Portugal

**Ing How Moo**

Singapore

**Shawnda Morrison**

Slovenia

**Stephen Muza**

United States Minor Outlying Islands

**David Nelson**

United States of America

**Eisuke Ochi**

Japan

**John Park**

United States of America

**Didier Payen**

France

**Xuetao Pei**

China

**Rui-yun Peng**

China

**Yang Peng**

United States of America

**Sandeep Phatak**

United States of America

**Ivan Poon**

Australia

**Georgi Popivanov**

Bulgaria

**Larry Pruitt**

United States of America

**Xing-Shun Qi**

China

**Wei-Min Qu**

China

**Daniel G. Remick**

United States of America

**Samaneh Rouhi**

Iran 

**Frank A. J. L. Scheer**

United States of America

**Alexa Smith-Osborne**

United States of America

**Mohan L. Sopori**

United States of America

**Michael Steketee**

United States of America

**Lei Su**

China

**Kentaro Sugano**

Japan

**Xin Sun**

China

**Zachary Taylor**

United States of America

**Stefania Tegli**

Italy

**Marcia Testa**

United States of America

**Gursimran Thandi**

United Kingdom

**James Tsai**

United States of America

**Jacob Turner**

United States of America

**Bhan Urvashi**

United States of America

**Timothy R. Varney **

United States of America

**Lakshmi Vas**

India

**Lee Vernon**

Singapore

**Michael Waller**

Australia

**De-yun Wang**

Singapore

**Fu-Sheng Wang**

China

**Guo-qiang Wang**

China

**Hong-ying Wang**

China

**Jiang Huai Wang**

Ireland

**Kuang-Te Wang**

Taiwan

**Yan-Jiang Wang**

China

**Yang Wang**

United States of America

**Bryant Webber**

United States of America

**Rodney Willoughby**

United States of America

**Mee Lian Wong**

Singapore

**Michael Wünning**

Germany

**Xuan Xiao**

China

**Tai Xie**

China

**Noriko Yaekashiwa **

Japan

**Xiao Yang**

China

**Zheng-lin Yang**

China

**Yong-Ming Yao**

China

**Xue Yu**

United States of America

**Jing Yuan**

China

**Paul Zarogoulidis**

Greece

**Lian-yang Zhang**

China

**Min Zhao**

United States of America

**Fei-Hu Zhou**

China

**Qi-Quan Zhou**

China

**Jure Zupan**

Slovenia

